# Moral Imagination: The Missing Component in Global Health

**DOI:** 10.1371/journal.pmed.0020400

**Published:** 2005-12-27

**Authors:** Solomon R. Benatar

## Abstract

Benatar explores the underlying reasons for our failure to make adequate progress in improving global health.

The deplorable state of global health and the failure to improve this state have been debated extensively. Recent editorials in the *Lancet* in relation to the failure of Roll Back Malaria and the potential failure of the 3 by 5 programme [1,2] illustrate how disappointment, surprise, and admonitions about such failures are usually followed by optimism about the success envisaged from future efforts [1,3].

There are several possible reasons for our failure to make adequate progress in improving global health. First, it seems that there is generally more interest in doing research to acquire new knowledge than in using existing knowledge, unless it is commercially profitable—illustrating how market forces are a more powerful influence on the practice of medicine than health needs [4]. Second, concern for those who are most severely affected by ill health seems to be generally transient, perhaps because they are anonymous and out of sight, but maybe also because their lives are less highly valued [5,6]. Third, there is a tendency to focus on new technologies through “silo” (narrowly contained) approaches to improving global health [7–9]. Fourth, there is insufficient attention to the social determinants of health [10,11].

Finally, while many are concerned about the plight of others, collective action through nongovernmental organisations can only achieve limited results, and there is reluctance to acknowledge and more explicitly address the indirect, causal, complex global system forces that underlie poverty and many fatal diseases [5,11–15]. Fortunately, there is now growing recognition that new infectious diseases pose a major threat to human health and security worldwide [16,17], and that imaginative new solutions are needed to improve global health [18,19]. [Fig pmed-0020400-g001]

**Figure pmed-0020400-g001:**
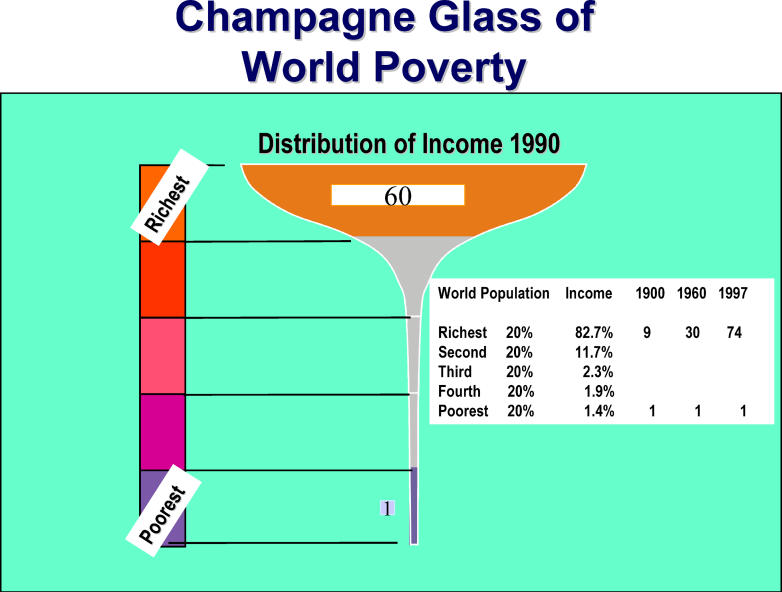
Global distribution of wealth (Figure adapted from [68])

While it is entirely appropriate to consider scientific and technological advances and economic growth as necessary for social progress, it is arguable that these will not be sufficient to ensure movement towards a more just world in which the health of whole populations could be improved. The controversy about globalisation versus antiglobalisation will not be revisited here, except to say that the debate should rather be about how globalisation can be modified to extend the benefits of progress more widely [20,21].

In this essay, I begin by suggesting that achieving substantial improvements in global health will depend on acknowledging that poor health at the level of whole populations reflects systemic dysfunction in a complex world. I then address why development aid is a necessary but not a sufficient solution for improving global health. I conclude with the idea that greater moral imagination (the ability of individuals and communities to empathise with others) and innovative 21st century approaches are required to break the impasse we currently face in improving global health.

## An Unstable and Dysfunctional World

In the domain of economics, there is a disjunction between massive economic growth over the past 50 years and fair distribution of new wealth [22]. The global economy has increased 7-fold since 1950, yet the disparity in per capita gross domestic product between the 20 richest and the 20 poorest nations has more than doubled between 1960 and 1995 [23]. As a result, there are ever-widening disparities between rich and poor (Figures 1 and 2), and almost half the world's population lives on less than US$2 per day [24]. Disproportionate pursuit of short-term self-interest, fostered by market fundamentalism, emphasises production of goods for consumption by individuals, corporations, and governments, while long-term interests and the production of public goods for whole populations are undervalued [25].

**Figure pmed-0020400-g002:**
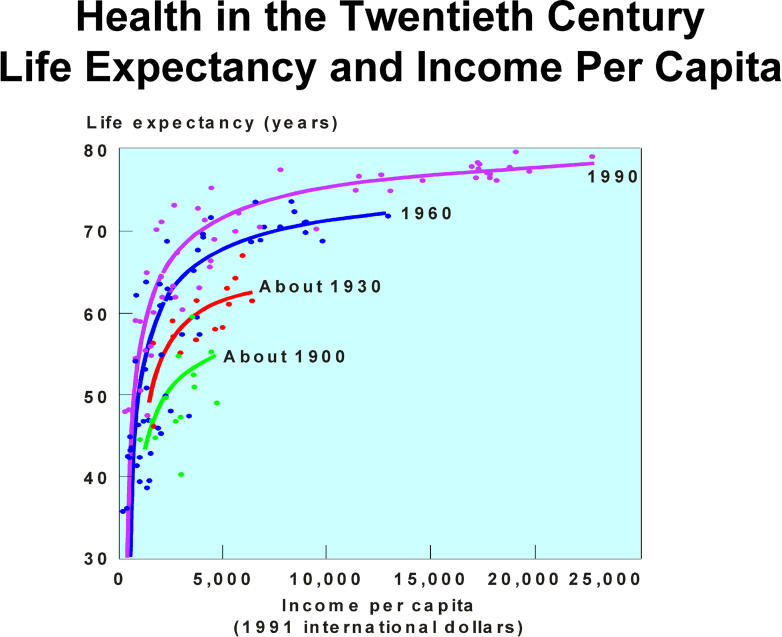
Relationship between life expectancy and income per capita (Figure adapted from [69])

Economic stability is threatened when aggregate economic growth is valued as an end in itself rather than as a means to improving human lives, and consequently, there is a failure to achieve a more just distribution of economic and social benefits [26]. Economic dysfunction persists when conventional economic theory continues to be revered and applied despite its many failures [26–29], and thus reduces the potential for improving global health and increasing human security worldwide [30].

In the domain of political and social life, instability is revealed by ongoing wars, ethnic conflict, fundamentalist attitudes, failed responses to genocide in many countries, large-scale disruption of communities, refugeeism, terrorism, fragmentation of health services, and attrition of public health-care services—all reflecting a lack of global leadership and a failure to achieve basic human rights for more people in the world [5,13,14,26,30,31]. Moreover, the full potential of the human rights approach is greatly diminished by a predominant focus on civil and political rights. Insufficient attention is paid to the social, cultural, and economic rights that are essential for human flourishing, and which are part of the “indivisible human rights” package described in the Universal Declaration of Human Rights, which most human rights activists use as their source of authority. It seems that higher value is placed on the rights and lives of those with resources than on the common good and the lives of the poor, and inadequate attention is given to identifying and motivating those who have duties to uphold a broad spectrum of rights [32,33].

These shortcomings, together with ecological instability from environmental degradation, global warming, and ongoing loss of biodiversity, arguably facilitate the creation of niches for the emergence and propagation of new infectious diseases, promote the development of multidrug resistance [34], and make it more difficult to maintain the social structures required to provide care and support for so many in need [35].

## Development Aid: A Necessary but Insufficient Solution

Greenwood's call for increased development aid to provide the US$2–US$5 needed for each year of life that could be saved through an effective worldwide malaria control programme [3] resembles the approaches taken for tuberculosis and HIV/AIDS (http://www.theglobalfund.org). It must be gratefully acknowledged that generous philanthropy from concerned individuals and many foundations, organisations, and new global initiatives can, and do, make valued contributions to improving the health and health care of marginalised people in the world. Development aid from many countries should also be welcomed, and recent endeavours to increase aid from the current average of 0.23% gross domestic product to the recommended 0.7% are admirable [36]. [Fig pmed-0020400-g002]

However, development aid has been progressively reduced in recent years, and is increasingly being directed towards emergency humanitarian aid and the perceived security needs of wealthy nations, rather than towards sustainable development [37,38]. Therefore, the main problem is not merely lack of philanthropy and development aid. More poignantly, the problem is how the high profile given to relatively small amounts of aid eclipses recognition of the fact that financial, human, and other material resources are continuously being extracted from developing countries by wealthy nations striving for their own ongoing economic growth [19,24].

Modern trade rules [39], bribery and other means of controlling national economies and the lives of millions of poor people [40], and recruitment of health professionals trained at the expense of developing countries to sustain health care in wealthy countries [41] all reflect new forms of exploitation that result in much more being extracted from developing countries than is given to them in aid or in any other form. For example, annual farming subsidies of US$350 billion in industrialised countries [42] and trade protectionism cost developing countries US$50 billion annually in export earnings [43]. Allowing farmers in developing countries to sell their products at a fair price and not in competition with massive subsidies could eliminate the need for development aid [39,44].

Debt is another major problem. Poor countries' debt (US$2.2 trillion in 1997) has been associated with, and perpetuated through, arms trading (often coercively linked to aid) [45–47]. Such debt, particularly sub-Saharan Africa's debt of US$275 billion, fostered by both eager lenders and often corrupt borrowers can never be repaid. Sustaining debt perpetuates economic dependence and human misery. Resulting annual interest payments, of greater magnitude than the US$21 billion annual aid donated to Africa, cripple health and other social services and stultify development [48,49].

While some countries have achieved economic development, this has been generally less than desired and, sadly, lacking in most of sub-Saharan Africa. Moreover, much done in the name of development has been counterproductive, with adverse effects on the potential for globally improved human security [50–53]. The meaning of development and its evaluation needs to be reconsidered. Development means more than overall economic growth, and must include social progress, for example, in basic living conditions, education, and access to health care, so that all can have the opportunity to reach their achievable human capacities [50,54,55].

The unpalatable facts about how development is stultified are not being adequately confronted, and little attempt is made to acknowledge and address the complex systemic forces that sustain poverty and poor health [19,24]. Instead, obfuscation by politicians and indomitable optimism focused on economics, science, and human rights all promote continued hope for improving health in the developing world through market forces and new technologies [7–9].

## Inadequate Moral Imagination

Some critical questions about world poverty have been asked and need to be answered [24]. Why does extreme poverty of almost half of humankind (income of less that US$2 per day) continue despite scientific, technological, economic, and moral progress? How do we explain why affluent individuals and wealthy nations are not morally embarrassed that so many people can be relegated to lives of poor quality with such limited opportunities to reach their full human potential? What does support (by individuals and nations) for processes that aggravate and sustain poverty tell us about ourselves and about the values we hold deeply? How can the rich remain secure in a world in which so many are so desperately poor that they may be provoked to rise up and rebel? Widening disparities within wealthy nations add another troubling dimension [56].

Many privileged people believe that poverty is not the fault of wealthy countries, but rather the result of bad government elsewhere. This is, indeed, partially true, and the prominent exposure of the extent of corruption and poor governance, for example in Africa [24,36,44,57], should be followed by sustained condemnation, retribution, and prevention. However, much less openly discussed is the complicity of powerful nations in supporting leaders who are despots and kleptocrats—by legitimising their right to sell their countries' natural resources, spend profligately on themselves, and incur debts that their impoverished citizens must repay [24,52]. Because wealthy nations, and by association their citizens, are deeply implicated in the generation and maintenance of forces that perpetuate social injustice and poverty, they need to face their responsibilities to alleviate the lives of those most adversely affected [24,52]. Reliance solely on perpetual philanthropy is clearly not the long-term solution to global health problems.

While we talk increasingly about disparities in wealth and health in an unjust world, most privileged people remain complacent about the suffering of the poor—both distant and within our midst [58,59]. In considering the many genocides across the world during the 20th century, Jonathan Glover has suggested that it is only moral imagination (our ability to imagine ourselves in the shoes of others) that can enable us to alter our outlook and actions significantly [60]. Our moral imagination is dulled, and insight into our global interdependence is diminished by insufficient public acknowledgment of how the quest of wealthy nations for endless economic growth, and luxuries that their citizens expect, has profoundly adverse effects on access to basic necessities of life for millions of others [24,26,49]. The ability to empathise with others requires the critical examination of our individual lives and of our nations' actions, the capacity to see ourselves as bound to all other human beings, and the sensitivity to imagine what it might be like to be a person living a very deprived and threatened life [24,61,62].

Making a diagnosis of social ills, like making diagnoses in medical practice, is much easier than providing effective remedies [63]. The magnitude and importance of achieving solidarity and cooperation in an interdependent world calls for a major research programme and considerable scholarship from many disciplines. Some pointers have been provided [18–21,24,64–66].

If lack of moral imagination were to be seen as one of the grand challenges for global health, resources and scholarly energy would surely be applied to promoting such imagination and to seeking innovative new approaches to improving global health. The quest for improved global health will be elusive if we continue to neglect the upstream forces that cause, sustain, and aggravate the poverty and misery that characterise the lives of almost half the world's population. The writing is on the wall [67].
